# The “gateway” effect of e-cigarettes may be explained by a genetic liability to risk-taking

**DOI:** 10.1371/journal.pmed.1003554

**Published:** 2021-03-18

**Authors:** Wayne Hall, Gary Chan

**Affiliations:** 1 National Centre for Youth Substance Use Research, The University of Queensland, Brisbane, Queensland, Australia; 2 Queensland Alliance for Environmental Health Sciences, The University of Queensland, Brisbane, Queensland, Australia

## Abstract

Wayne Hall and Gary Chan discuss Jasmine Khouja and co-workers’ accompanying study on genetic susceptibility to tobacco smoking initiation and e-cigarette use.

E-cigarettes have become a popular method of smoking cessation and a long-term reduced-harm alternative to tobacco smoking in countries that allow their sale [1]. There is reasonable evidence of their effectiveness for smoking cessation [2] and their value in reducing harm for smokers [1,2], but critics have argued that e-cigarettes are likely to harm public health by serving as a gateway to cigarette smoking in young adults [3,4]. In Australia, concern about this putative gateway effect has led health regulators to ban the sale of e-cigarettes to adult smokers without a medical prescription [5]. In surveys, most adolescents and young adults who have used e-cigarettes have smoked cigarettes (although fewer have done so before than after smoking) [6]. Some authors have seen these findings as evidence that e-cigarettes are a gateway to smoking [7], but others argue that they are better explained by a liability to engage in risky behaviour that makes some young people more likely to smoke conventional cigarettes and to try e-cigarettes [6,8].

In this issue of *PLOS Medicine*, Jasmine Khouja and colleagues report one of the first studies to assess whether shared genetic risk factors may explain the association between e-cigarette use and cigarette smoking. They used data from a very large longitudinal cohort study in the United Kingdom, the Avon Longitudinal Study of Parents and Children, of young adults aged 23 to 26 years and calculated 5 polygenic risk scores (PRSs) for smoking initiation that they derived from genome-wide association studies of smoking initiation. They used logistic regression to assess whether the association between self-reported smoking initiation and e-cigarette use was explained by the PRS for smoking initiation. They also included negative control analyses to assess whether these PRSs were associated with other established risk factors for smoking in young people, namely, socioeconomic position at birth, externalising disorders in childhood, and risk-taking in young adulthood.

Khouja and colleagues found similar associations between the smoking initiation PRS and the initiation of both cigarette smoking (odds ratio [OR] 1.29, 95% CI 1.19 to 1.39) and e-cigarette use (OR 1.24, 95% CI 1.14 to 1.34) by the age of 24 and an association between the PRS for smoking initiation and e-cigarette use in never smokers. The PRS for smoking initiation was also associated with the risk of gambling, a larger number of sexual partners, conduct disorder at 7 years, and parental socioeconomic position at birth. Khouja and colleagues argue that their results indicate that there may be a shared genetic aetiology for cigarette smoking and e-cigarette use and for socioeconomic position, externalising disorders in childhood, and risky behaviour. Taken together, their findings suggest that smoking and e-cigarette use are both reflections of a broad risk-taking phenotype.

No single study is ever decisive, but Khouja and colleagues’ findings are consistent with other epidemiological evidence. This includes findings that the adolescents who are most likely to experiment with e-cigarettes are those who are at higher risk of smoking cigarettes (and using other drugs) because of traits such as sensation seeking, risk-taking, and oppositional behaviour [8,9]. E-cigarette use has also not been accompanied by increased cigarette smoking among young people in the United States [10], as would be the case if e-cigarette use were a major gateway to cigarette smoking [8,9,11]. The latter findings suggest that any gateway effect of e-cigarettes is small at the population level because smoking prevalence has continued to decline, despite an increased uptake of e-cigarettes among young adults in countries that allow their sale [6,8,10].

It is still prudent public health policy to minimise youth uptake of e-cigarettes, even if their use does not lead to cigarette smoking. However, this does not require a sales ban on nicotine vaping products to adult smokers. More proportionate regulatory policies that have reduced the uptake of cigarette smoking among youth could also minimise youth uptake of e-cigarettes, namely, setting age limits on purchase, restricting the number and types of outlets where e-cigarettes can be sold, and prohibiting the marketing and advertising of e-cigarettes. These policies would allow adult smokers to access e-cigarettes for smoking cessation or as a lower risk substitute for cigarette smoking [6].

## References

[1] McNeill A, Brose LS, Calder R, Bauld L, Robson D. Evidence review of e-cigarettes and heated tobacco products 2018. A report commissioned by Public Health England London: Public Health England. 2018;6. Available from: https://assets.publishing.service.gov.uk/government/uploads/system/uploads/attachment_data/file/684963/Evidence_review_of_e-cigarettes_and_heated_tobacco_products_2018.pdf

[2] Hartmann-Boyce J, McRobbie H, Lindson N, Bullen C, Begh R, Theodoulou A, et al. Can electronic cigarettes help people stop smoking, and do they have any unwanted effects when used for this purpose? Cochrane Database Syst Rev. 2020;10. Available from: https://www.cochranelibrary.com/cdsr/doi/10.1002/14651858.CD010216.pub4/full 10.1002/14651858.CD010216.pub4 PMC8094228 33052602

[3] McKee M. Evidence and e-cigarettes: explaining English exceptionalism. Am J Public Health. 2019;109(7):965–6. 10.2105/AJPH.2019.305132 31166720 PMC6603487

[4] Chapman S, Bareham D, Maziak W. The gateway effect of e-cigarettes: reflections on main criticisms. Nicotine Tob Res. 2019;21(5):695–8. 10.1093/ntr/nty067 29660054 PMC6468127

[5] Australian Government Department of Health. Submission 167 to the Select Committee on Tobacco Harm Reduction. 2020. Available from: https://www.aph.gov.au/Parliamentary_Business/Committees/Senate/Tobacco_Harm_Reduction/TobaccoHarmReduction/Submissions

[6] Mendelsohn CP, Hall W. Does the gateway theory justify a ban on nicotine vaping in Australia? Int J Drug Policy. 2020;78:102712. 10.1016/j.drugpo.2020.102712 32145594

[7] National Academies of Sciences Engineering and Medicine. Public health consequences of e-cigarettes. National Academies Press; 2018. 29894118

[8] Chan GCK, Stjepanovic D, Lim C, Sun T, Anandan AS, Connor J, et al. Gateway or common liability? A systematic review and meta-analysis of studies of adolescent e-cigarette use and future smoking initiation. Addiction. 2020 9 4. 10.1111/add.15246 32888234

[9] Kozlowski LT, Warner KE. Adolescents and e-cigarettes: objects of concern may appear larger than they are. Drug Alcohol Depend. 2017;174:209–14. 10.1016/j.drugalcdep.2017.01.001 29350617

[10] Miech R, Johnston L, O’Malley P, Bachman J, Schulenberg J, Patrick M. Monitoring the Future national survey results on drug use 1975–2019: Overview, key findings on adolescent drug use. Ann Arbor: Institute for Social Research, University of Michigan; 2020.

[11] Glasser A, Johnson A, Niaura R, Abrams D, Pearson J. Youth vaping and tobacco use in context in the United States: results from the 2018 National Youth Tobacco Survey. Nicotine Tob Res. 2020 1 13. 10.1093/ntr/ntaa010 31930295

